# The synthetic estradiol analog E0703 enhances *Akkermansia muciniphila* growth for radiation‐induced intestinal damage repair

**DOI:** 10.1002/mlf2.70071

**Published:** 2026-04-30

**Authors:** Zhexin Ni, Ziqiao Yan, Mingyang Chang, Yangshuo Li, Zebin Liao, Tiantian Xia, Zhijie Bai, Ningning Wang, Chaoji Huangfu, Dezhi Sun, Yangyi Hu, Liangliang Zhang, Feiran Hao, Yongqi Dou, Pan Shen, Wei Zhou, Yue Gao

**Affiliations:** ^1^ Beijing Institute of Radiation Medicine Beijing China; ^2^ Department of Traditional Chinese Medicine The Sixth Medical Center of Chinese People's Liberation Army (PLA) General Hospital Beijing China; ^3^ Chinese PLA Medical School, Chinese People's Liberation Army (PLA) General Hospital Beijing China; ^4^ Department of Traditional Chinese Gynecology The First Affiliated Hospital of Naval Medical University Shanghai China; ^5^ Medical College of Qinghai University Xining China; ^6^ School of Pharmacy Guangdong Pharmaceutical University Guangzhou China; ^7^ State Key Laboratory of Kidney Diseases, Chinese PLA General Hospital Beijing China

**Keywords:** acute radiation, *Akkermansia muciniphila*, E0703, goblet cells

## Abstract

The development of safe and effective radioprotective agents with minimal side effects, particularly for high‐dose exposure, remains a global priority. E0703, a novel steroidal compound structurally derived from estradiol, has shown promising radioprotective efficacy with limited estrogenic activity in prior pharmacodynamic studies. In this study, E0703 was found to significantly increase the abundance of *Akkermansia muciniphila* (AKK) in the intestines of both irradiated and non‐irradiated mice. Co‐administration of E0703 and AKK markedly improved the 7‐day survival rate of mice exposed to a lethal 8.5 Gy dose of radiation. E0703 induced beneficial transcriptional changes in AKK, with enrichment in metabolic pathways such as amino acid biosynthesis, aminoacyl‐tRNA biosynthesis, the tricarboxylic acid (TCA) cycle, and fatty acid biosynthesis. These alterations supported the production of glucosamine 6‐phosphate (GlcN‐6‐P) by AKK, which contributed to intestinal tissue regeneration following irradiation. Single‐cell transcriptomic analysis revealed that E0703 significantly increased the proportion of intestinal stem cells and goblet cells by Day 5 post irradiation. Mechanistically, E0703 modulated the oxidative phosphorylation pathway in these cell types, including regulation of Muc2 production. E0703 also enhanced AKK abundance in irradiated mice, particularly in the presence of mucin, thereby elevating the availability of GlcN‐6‐P—a critical substrate for intestinal organoid repair. These findings indicate that E0703 exerts direct effects on goblet cells and AKK, promoting host–microbe interactions that facilitate intestinal regeneration and improve survival following radiation exposure.

## Impact statement

Developing safe and effective radioprotective agents with minimal side effects remains a significant challenge, especially for high‐dose radiation exposure. This study identifies E0703 as a novel radioprotective agent that promotes the expansion of *Akkermansia muciniphila* (AKK) in the gut. The combined effects of E0703 and AKK significantly enhance survival in mice exposed to lethal radiation. E0703 supports intestinal repair by modulating host cell activity and enhancing beneficial microbial metabolism. These findings offer a promising strategy for improving radioprotection in medical and emergency settings.

## INTRODUCTION

In recent years, global concern over the toxicities associated with radiation exposure has grown markedly. Radiation therapy, though highly effective for targeting and destroying cancer cells, unavoidably also affects surrounding healthy tissues, leading to a variety of radiation‐induced injuries^1^. These adverse effects include not only physical harm but also psychological distress, including increased anxiety, higher rates of disease, and mortality^2^, ^3^. Radiation exposure, whether from therapeutic applications or from accidental sources such as industrial incidents or nuclear disasters, poses significant threats to human health. With growing awareness of nuclear safety and continual advances in clinical radiotherapy, interest has increased in developing agents that can protect against the detrimental effects of nuclear radiation^4^, ^5^, ^6^.

Estrogens were first reported in the early 1990s as having protective properties against radiation injury (RI)^7^, ^8^, stimulating extensive research in this field^9^, ^10^, ^11^. Compounds such as estradiol, estriol, and nilestriol have been shown to provide prophylactic effects when administered before or shortly after radiation exposure, facilitating rapid recovery of radiation‐sensitive tissues. These agents also confer extended periods of protection. However, their efficacy diminishes at higher radiation doses, and they frequently cause undesirable estrogenic side effects^12^, ^13^. The synthetic steroidal compound E0703, structurally derived from estradiol, has shown promising radioprotective effects with minimal estrogenic side effects in preliminary pharmacodynamic studies. Data indicated that pre‐irradiation administration of E0703 significantly improves survival and enhances white blood cell counts, including leukocytes, neutrophils, and granulocytes. Moreover, E0703 stimulates the proliferation of hematopoietic stem cells, as demonstrated in murine and canine models^14^, ^15^, ^16^, ^17^. Although E0703 appears to be a promising pharmaceutical agent for mitigating the harmful effect of radiation, the precise biochemical mechanisms underlying its radioprotective activity after oral administration remain unclear.

The gut microbiota and their metabolites play critical roles in regulating host metabolism, modulating immune responses, and maintaining homeostasis within the internal environment^18^. It is well established that radiation therapy profoundly alters the gut microbiota, leading to dysbiosis—an imbalance characterized by reduced microbial diversity. Such dysbiosis is associated with increased inflammation, elevated oxidative stress, and subsequent tissue injury. Notably, RI represents a more severe clinical challenge than previously appreciated, with the pathological features of radiation‐induced intestinal injury resembling those of inflammatory bowel disease, but occurring with greater frequency. Up to 90% of individuals undergoing abdominal or pelvic radiation therapy are estimated to experience gastrointestinal symptoms during the first weeks of treatment^19^. This highlights the urgent need for effective therapies for RI, and suggests that manipulation of the gut microbiota may provide a promising therapeutic approach.

In this study, we examined the potential of E0703 to mitigate acute RI in mouse models. Our results demonstrated that a single oral administration of E0703 provided protection against gastrointestinal toxicity induced by 8.5 Gy radiation exposure. Mechanistically, we found that E0703 treatment promoted Muc2 protein expression and increased the abundance of *Akkermansia muciniphila* (AKK) following irradiation. Furthermore, the oxidative phosphorylation pathway in stem and goblet cells, as well as the glucosamine 6‐phosphate (GlcN‐6‐P) produced by AKK, appeared to be central to the radioprotective effects of E0703. Collectively, our research advances understanding of microbiota‐based E0703 intervention as a preventative strategy for RI.

## RESULTS

### E0703 improves the weight and survival rate of irradiated mice

**Figure 1:**
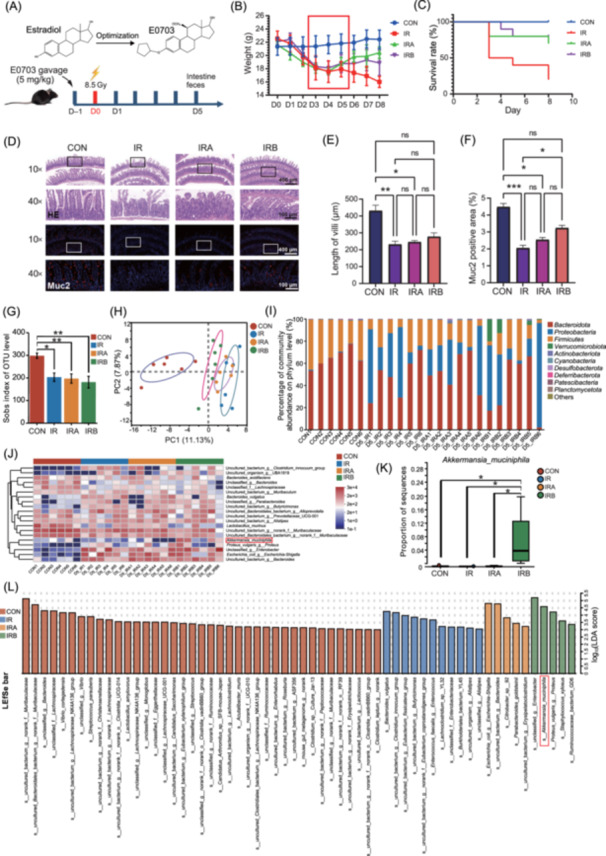
E0703 enhances survival and intestinal repair following irradiation in mice. (A) Structure of E0703 and schematic diagram of animal intervention. E0703, a structural analog of estrogen, was administered to mice at 5 mg/kg 24 h prior to irradiation. Intestinal tissues and fecal samples were collected on Day 5 (D5) post irradiation. (B) Body weight analysis of different groups of mice 1 week after irradiation. One week after irradiation, body weight in the irradiated (IR) group declined significantly, whereas Amifostine (IRA) and E0703 (IRB) groups demonstrated weight recovery from Day 4 to Day 5. (C) Survival analysis of mice 1 week after irradiation. The survival rate in the IR group decreased markedly within 1‐week post irradiation, while IRA and IRB groups maintained approximately 80% survival. (D) Histopathological results of the small intestine on Day 5 after irradiation. Irradiation disrupted small intestine structure, reduced villus number and length, and decreased the fluorescence intensity of the mucin protein Muc2. Both Amifostine and E0703 preserved villus structure and Muc2 expression, with E0703 showing a more pronounced effect. Scale bars: 100 μm and 400 μm. (E, F) Quantitative analysis of small intestinal pathology (E) and Muc2 expression (F). A trend toward improved villus length with E0703 is shown, though this is not statistically significant. E0703 significantly enhanced Muc2 expression compared with the IR group (*p* < 0.05), showing greater efficacy than Amifostine. (G) Sobs index analysis of intestinal microbiota in mice on Day 5 after irradiation. Radiation significantly reduced gut microbiota richness, with no significant improvement in drug‐treated groups. (H) PCA analysis of intestinal microbiota in mice on Day 5 after irradiation. Beta diversity analysis demonstrated distinct clustering among groups, with IRB and CON groups showing the closest distance. (I) Phylum‐level composition of intestinal microbiota in mice on Day 5 after irradiation. Radiation caused significant changes in gut microbiota composition, notably an increased abundance of *Verrucomicrobiota* in the IRB group. (J) Species‐level composition of intestinal microbiota in mice on Day 5 after irradiation. At the species level, *Akkermansia muciniphila* (AKK) abundance was significantly higher in the IRB group. (K) Comparison of intestinal AKK abundance in mice on Day 5 after irradiation. E0703 significantly increased AKK in irradiated mice (*p* < 0.05). (L) LEfSe analysis of intestinal microbiota in mice on Day 5 after irradiation. Significant microbial groups was identified, with higher LDA scores indicating a greater effect of species abundance. CON, nonirradiated mice; LDA, linear discriminant analysis; LEfSe, LDA effect size; OTU, operational taxonomic unit; PCA, principal component analysis. Group differences were evaluated using one‐way analysis of variance (ANOVA). ns, not significant. *p < 0.05; **p < 0.01; and ***p < 0.001.

E0703, a compound derived from estradiol, shows significant radioprotective properties through selective activation of estrogen receptor 2 (Esr2) (Figures 1A and S1A,B). In this study, mice exposed to whole‐body irradiation (8.5 Gy) experienced substantial weight loss (Figure 1B) and reduced survival rates, declining to 50% on Day 3 and 20% on Day 8 post irradiation (Figure 1C). Amifostine, serving as a positive control, was administered intraperitoneally (150 mg/kg) 0.5 h before irradiation (IRA group), whereas E0703 was administered orally to the IRB group (5 mg/kg) 24 h prior to irradiation (Figure 1A). Both IRA and IRB groups showed the lowest body weight on Day 4 post irradiation, followed by a progressive increase, with no significant difference between groups observed on Day 8 (Figure 1B). Survival in the IRA group reached 80% on Day 3 and 70% on Day 8, whereas the IRB group demonstrated 100% survival on Day 3 and 80% survival on Day 5 and 7 (Figure 1C). These results suggest a pivotal recovery phase between Day 4 and 5 after irradiation, as indicated by the observed weight gain in surviving mice, likely reflecting a reparative response to radiation‐induced intestinal injury. Radiation has a detrimental effect on the sensitive villi of the small intestine, where preservation of the mucus layer is essential for restoring intestinal barrier function after injury. To determine the cause of the weight gain observed in IRA group mice on Day 5 post irradiation, we performed histopathological analysis of small intestinal tissues (Figure 1D). Villi length was markedly reduced in irradiated mice compared to nonirradiated controls. Neither Amifostine nor E0703 significantly restored villus length on Day 5 post irradiation (Figure 1E). However, Muc2 expression level in the E0703‐treated group was significantly greater than that in the irradiated‐only group (*p* < 0.05, Figure 1F). In addition, E0703 promoted the growth of intestinal organoids after irradiation, and this effect was abrogated by an Esr2 inhibitor (Figure S1C,D). These findings indicate that the radioprotective effect of E0703 may be attributable to enhanced intestinal Muc2 secretion.

### E0703 induces an increase in *A. muciniphila* abundance in irradiated mice

The gut microbiota plays a critical role in repairing the intestinal barrier and maintaining intestinal homeostasis. On Day 5 following 8.5 Gy whole‐body irradiation, we analyzed the fecal microbiota of each group using 16S rDNA gene sequencing. Irradiation not only compromised the intestinal barrier but also markedly reduced gut microbial diversity (Figure 1G), leading to substantial divergence from the microbiota of nonirradiated controls (Figure 1H). Although E0703 did not fully restore microbial diversity, it lessened the disparity with the control group's microbiota. At the phylum level, irradiation increased the abundance of *Proteobacteria* and decreased *Bacteroidota* and *Firmicutes* on Day 5 (Figure 1I). There were no significant changes in the phylum‐level composition in mice treated with Amifostine compared with those receiving irradiation alone. Notably, the proportion of *Verrucomicrobiota* was significantly elevated in the E0703 group (Figure 1I). At the species level, AKK, belonging to the *Verrucomicrobiota*, was substantially more abundant in the E0703 group than in the other groups (Figures 1J,K and S1E). Linear discriminant analysis effect size (LEfSe) confirmed AKK as a significant intergroup differentiator post irradiation in the E0703 group, with log_10_(LDA score) exceeding 4.5, (LDA, linear discriminant analysis), indicating that E0703 robustly induces AKK proliferation in irradiated mice (Figure 1L).

**Figure 2:**
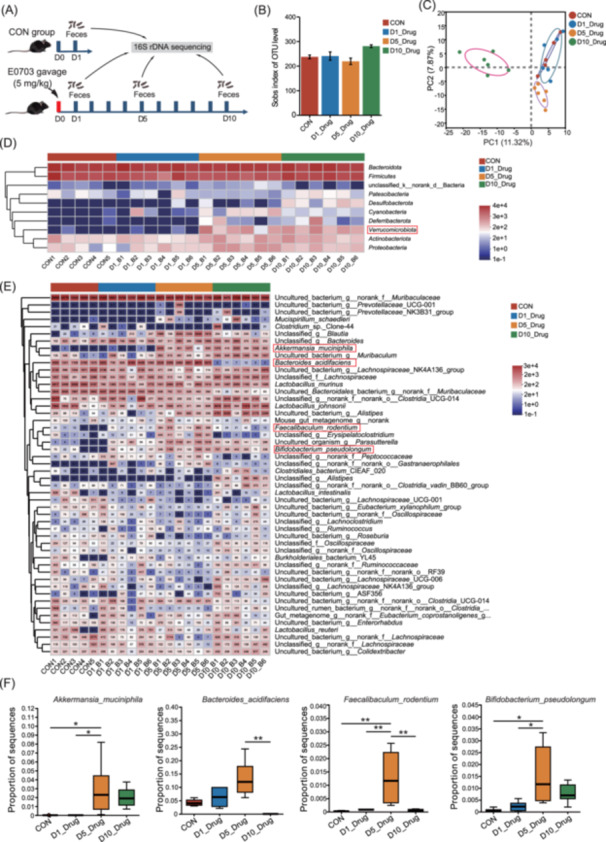
E0703 increases AKK abundance in the mouse intestine. (A) Schematic diagram of fecal microbiota sampling after E0703 intervention under nonirradiated conditions. Fecal samples from E0703‐treated normal mice were collected on Day 1, 5, and 10 for 16S rDNA sequencing. (B) Sobs index analysis of intestinal microbiota at different time points after E0703 intervention. A nonsignificant trend toward increased richness following E0703 treatment is shown. (C) PCA analysis of intestinal microbiota at different time points after E0703 intervention. E0703 altered gut microbiota structure over time. (D) Phylum‐level composition of intestinal microbiota at different time points after E0703 intervention. The relative abundance of *Verrucomicrobiota* increased significantly on Day 1, 5, and 10. (E) Species‐level composition of intestinal microbiota at different time points after E0703 intervention. E0703 significantly modified species‐level microbiota composition over time, increasing beneficial bacteria such as AKK, *Bacteroides acidifaciens*, *Faecalibaculum rodentium*, and *Bifidobacterium pseudolongum*. (F) Comparative analysis of the abundance of four probiotics at different time points after E0703 intervention. Single‐dose E0703 administration more effectively increased the abundance of these probiotics on Day 5 than on Day 10. Group differences were evaluated using one‐way analysis of variance (ANOVA). *p < 0.05; **p < 0.01.

As AKK is recognized as a potent probiotic for intestinal repair, we hypothesized that E0703 may enhance the survival rate of mice during the first week following 8.5 Gy irradiation by stimulating AKK proliferation. To assess the temporal effect, we examined gut microbiota composition on Day 1, 5, and 10 after E0703 administration (Figure 2A), and found no significant changes in overall biodiversity (Figure 2B). Principal component analysis (PCA) revealed substantial shifts in the gut microbiota after E0703 treatment (Figure 2C). On Day 5, the phylum *Verrucomicrobiota* was notably enriched (Figure 2D). Detailed analysis indicated that E0703 increased the abundance of several beneficial bacteria, including AKK, *Bacteroides acidifaciens*, *Faecalibaculum rodentium*, and *Bifidobacterium pseudolongum* (Figure 2E). In particular, AKK abundance peaked on Day 5, exceeding all other time points (Figure 2F), highlighting the capacity of E0703 to promote beneficial gut bacteria in the context of irradiation.

### Combination of *A. muciniphila* and E0703 improves intestinal damage in irradiated mice

**Figure 3:**
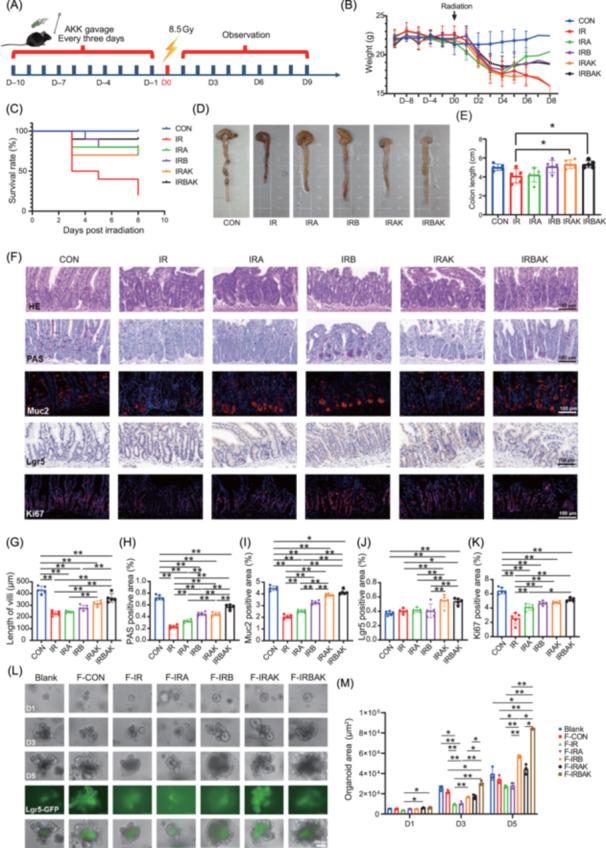
AKK supplementation potentiates E0703‐mediated radiation resistance and intestinal protection. (A) Schematic diagram of AKK intervention in irradiated mice. Mice received AKK by gavage every 3 days for 10 days prior to irradiation. Intestinal tissues and fecal samples were collected on Day 5 post irradiation. (B) Body weight analysis of mice 1 week after irradiation under different drug interventions. AKK gavage did not affect body weight before irradiation but improved recovery in irradiated mice on Day 4 and 5. (C) Survival analysis of mice 1 week after irradiation under different drug interventions. AKK alone enhanced survival after irradiation, while its combination with E0703 maintained 90% survival. (D, E) Colon length and its quantification on Day 5 after irradiation under different drug interventions. Irradiation reduced rectal length, but AKK or E0703, alone or combined, improved rectal length (*p* < 0.05). (F–K) Histopathology of the small intestine and quantitative analysis on Day 5 after irradiation under different drug interventions. Histological analysis showed that AKK, E0703, and their combination improved villus length, PAS‐positive area, and fluorescence area proportions of Muc2, Lgr5, and Ki67, with the combination group showing the best effect. Scale bar: 100 μm (F). (L, M) Representative images and quantification of intestinal organoid intervention with fecal supernatants. Fecal supernatants from treated mice promoted intestinal organoid growth, with the combination group demonstrating the most pronounced effect. IRAK, AKK‐treated; IRBAK, AKK‐, and E0703‐treated before irradiation. F, fecal supernatant; PAS, Periodic acid‐Schiff. Group differences were evaluated using one‐way analysis of variance (ANOVA). *p < 0.05; and **p < 0.01. Scale bar: 100 μm (L).

To assess the therapeutic potential of AKK in radiation‐induced intestinal injury, we conducted bacterial transplantation experiments. In addition to the CON, IR, IRA, and IRB groups, two new groups were established: the IRAK group, in which AKK was administered by gavage 10 days before irradiation, and the IRBAK group, in which AKK intervention was combined with E0703 administered orally 24 h prior to irradiation (Figure 3A). The results showed that groups IRA, IRB, and IRBAK all experienced increases in average body weight 1 week after irradiation, with no significant difference among these groups on Day 7 (Figure 3B). Notably, AKK gavage alone (IRAK group) maintained a 70% survival rate within 7 days post irradiation. The combination (IRBAK group) resulted in the highest survival rate (90%) within the same period, exceeding the 80% observed in both IRA and IRB groups (Figure 3C). Additionally, both IRAK and IRBAK were effective in promoting colon length recovery after irradiation (*p* < 0.05) (Figure 3D,E).

Radiation exposure resulted in considerable damage to the intestinal epithelium and goblet cells, including marked reductions in villus length and in Periodic acid‐Schiff (PAS)‐ and Muc2‐positive areas (*p* < 0.05). Co‐administration of E0703 and AKK significantly ameliorated this injury, restoring villus length and increasing PAS and Muc2 positivity to a greater extent than E0703 or AKK alone (*p* < 0.05) (Figure 3F–I). Regeneration of the intestinal epithelium after radiation depends largely on intestinal stem cells (ISCs). We therefore assessed the expression of ISC marker genes *Lgr5* and *Olfm4*. Surprisingly, there was no significant difference in Lgr5 and Olfm4 expression between the CON and IR groups (*p* > 0.05) (Figures 3F and S1I). This may reflect the timing of sampling, as on Day 5 post irradiation, the intestine may be in a recovery phase with normalized ISC levels following an initial decline. However, the combination of E0703 and AKK significantly elevated Lgr5 and Olfm4 expression beyond that of non‐irradiated controls (*p* < 0.05) (Figures 3J and S1F), suggesting that this combination therapy may robustly promote crypt cell repopulation during recovery.

Radiation‐induced suppression of intestinal cell proliferation was evidenced by a significant reduction in the Ki67‐positive area (Figure 3K) and a significant increase in the terminal deoxynucleotidyl transferase dUTP nick end labeling (TUNEL)‐positive area (Figure S1G,I), indicating decreased proliferative capacity (*p* < 0.05). Combined administration of E0703 and AKK counteracted these effects (*p* < 0.05) (Figures 3K and S1G), highlighting the efficacy of this approach in supporting intestinal regeneration post radiation. In addition, we evaluated intestinal barrier lysozyme expression, which is essential for defense against microbial translocation. Radiation exposure caused a pronounced decrease in the Lyz‐positive area (*p* < 0.05), which was repaired by combined E0703 and AKK administration (Figure S1H).

Intestinal organoids were utilized as an *in vitro* model to assess the effect of fecal supernatants from the different groups on organoid growth, compared to standard organoid medium as a blank control. Fecal supernatants from the IRB (F‐IRB) and IRBAK (F‐IRBAK) groups significantly promoted organoid growth and increased organoid area on Day 3 and 5 of culture compared to F‐IR and F‐IRA (*p* < 0.05) (Figure 3L,M). Fecal supernatant from the IRAK group (F‐IRAK) also enhanced organoid growth, but to a lesser extent than F‐IRB and F‐IRBAK (Figure 3M). In addition, organoids in the F‐IRAK and F‐IRBAK groups showed higher ZO‐1 expression than that in the F‐IR, F‐IRA, and F‐IRB groups (Figure S1J), suggesting an important role for AKK in intestinal barrier formation.

### E0703 promotes the production of glucosamine‐6‐phosphate by AKK to repair radiation‐induced damage in intestinal organoids

**Figure 4:**
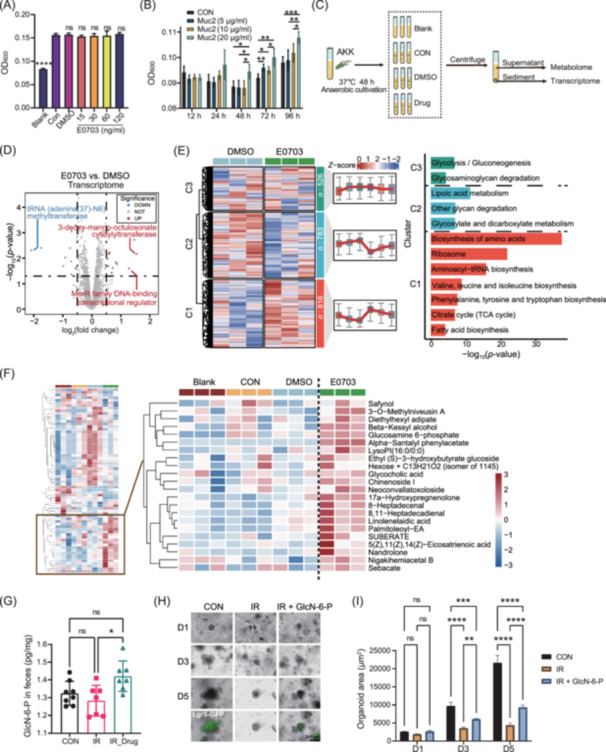
E0703 modulates the transcriptome and metabolome of AKK. (A) OD_600_ analysis of AKK after 48 h E0703 intervention *in vitro*. Optical density (OD) measurements showed no significant effect of E0703 on AKK growth. (B) OD_600_ analysis of AKK from 12 to 96 h after Muc2 intervention *in vitro*. Recombinant human Muc2 protein enhanced AKK growth in a dose‐ and time‐dependent manner. (C) Schematic diagram of supernatant metabolomics and pellet transcriptomics after a 48 h E0703 intervention in AKK *in vitro*. Blank, normal medium; CON, AKK without intervention; DMSO, AKK with dimethyl sulfoxide; and Drug, AKK with E0703 in DMSO. (D) Volcano plot comparing gene expression between E0703 and DMSO groups (*p* < 0.05, log_2_(fold change) >0.5). (E) Heatmap of differentially expressed genes clustered by M‐fuzz, with KEGG enrichment shown. (F) Heatmap of significantly different metabolites, highlighting those with higher abundance in the E0703 group. (G) Comparison of GlcN‐6‐P levels in mice feces across groups. (H, I) Representative images and quantitative analysis of intestinal organoids treated with GlcN‐6‐P. GlcN‐6‐P intervention improved the growth of irradiated intestinal organoids (*p* < 0.05). Group differences were evaluated using one‐way analysis of variance (ANOVA). ns, not significant; *p < 0.05; **p < 0.01; ***p < 0.001; and ****p < 0.0001. Scale bar: 200 μm (H).

The above results demonstrate that E0703 increases *Akkermansia* abundance *in vivo*. To determine whether E0703 directly stimulates AKK proliferation, we performed an *in vitro* culture of AKK with E0703 for 48 h. E0703 did not significantly promote AKK proliferation *in vitro* (Figure 4A). Since AKK relies on intestinal mucin as its primary nutrient, and E0703 effectively increased Muc2 expression in the irradiated intestine (Figure 1F), we tested whether Muc2 protein promotes AKK proliferation. Indeed, Muc2 protein markedly increased AKK growth (Figure 4B). To further characterize the interaction, we performed transcriptomic and metabolomic profiling of AKK and its culture supernatant (Figure 4C). E0703 altered the transcriptome of AKK, with significant upregulation of genes encoding proteins such as 3‐deoxy‐manno‐octulosonate cytidylyltransferase and MerR family DNA‐binding transcriptional regulator compared to the DMSO control (Figure 4D). Kyoto Encyclopedia of Genes and Genomes (KEGG) enrichment analysis identified significant enrichment in pathways, including amino acid biosynthesis, aminoacyl‐tRNA biosynthesis, the citrate (TCA) cycle, and fatty acid biosynthesis, in the E0703 group (Figures 4E and S2A). Metabolomic analysis showed that E0703 altered AKK metabolic profiles, notably increasing GlcN‐6‐P in glycolysis (Figures 4F and S2B). Elevated GlcN‐6‐P levels were also detected in feces from the E0703‐treated group (Figure 4G), and GlcN‐6‐P was shown to promote the growth of irradiated intestinal organoids (Figure 4H,I). These data indicate that E0703 can modify AKK transcriptional and metabolic activity, with AKK‐derived GlcN‐6‐P contributing to intestinal recovery after radiation exposure.

'Collectively, these findings support the hypothesis that E0703 increases AKK abundance in the murine intestine indirectly, by promoting Muc2 secretion, rather than by directly stimulating AKK proliferation. Mucin, secreted by intestinal goblet cells, is a critical nutrient for AKK. The above results demonstrated that E0703 administration upregulated Muc2 expression in irradiated mice (Figure 1, 3), potentially creating an environment favorable for AKK proliferation and thereby promoting intestinal barrier repair after radiation. The precise mechanisms by which E0703 induces Muc2 upregulation remain to be determined.

### E0703 promotes stem cell recovery and goblet cell production of Muc2 through the oxidative phosphorylation pathway

**Figure 5:**
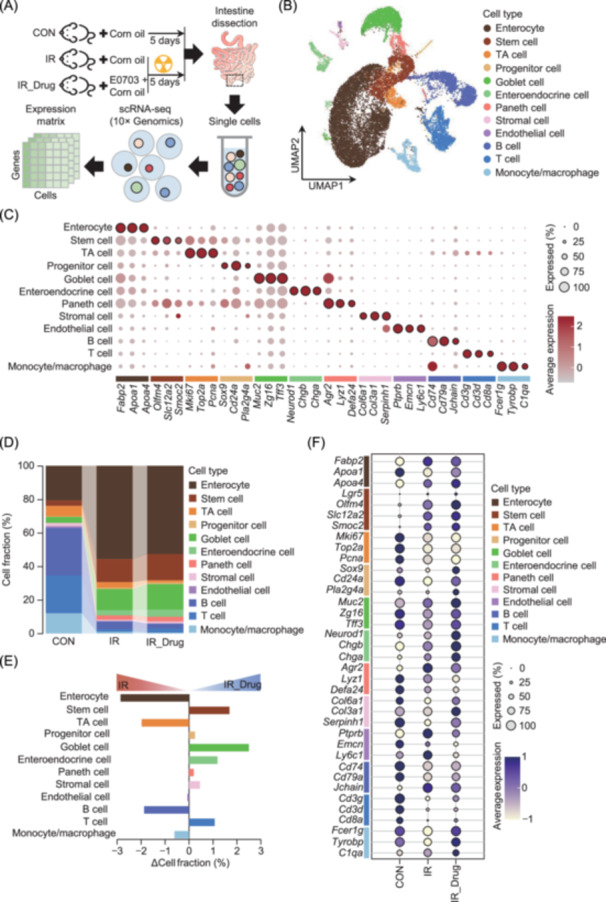
Single‐cell RNA sequencing (scRNA‐seq) reveals the protective effect of E0703 in radiation‐induced intestinal injury. (A) Schematic of the scRNA‐seq experimental design. (B) Single‐cell atlas of mouse intestines following irradiation and E0703 treatment. (C) Dot plot of normalized mean expression of canonical marker genes across clusters, with dot size indicating the proportion of expression. Average expression denotes the mean expression level of a gene across diverse cell populations, with darker colors indicating higher expression levels. Expressed (%) illustrates the percentage of cells within a particular cell population that expresses the specified gene. (D) Alluvial chart showing cell type proportions in control, IR, and E0703‐treated groups. (E) Changes in cell proportions 5 days post irradiation and after E0703 treatment. ΔCell fraction (%) means the difference in cell fraction between IR_Drug and IR groups. (F) Dot plot of marker gene expression across clusters and conditions, with dot size indicating expression proportion.

**Figure 6:**
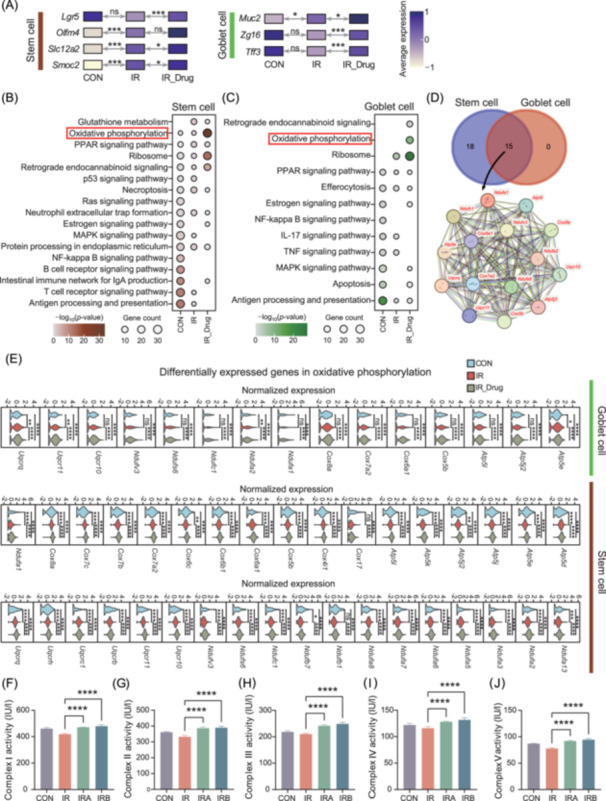
E0703 enhances oxidative phosphorylation, increasing intestinal stem and goblet cell ratios after irradiation. (A) Heatmap illustrating normalized mean expression of marker genes in stem and goblet cells. (B, C) Dot plots of Kyoto Encyclopedia of Genes and Genomes (KEGG) pathway enrichment in stem and goblet cells across conditions. Darker colors indicate smaller *p*‐values and larger dots indicate more genes. (D) Genes commonly enriched in the oxidative phosphorylation pathway in both cell types. (E) Gene expression comparison in the oxidative phosphorylation pathway across groups. (F–J) Both Amifostine and E0703 improved mitochondrial complex I–V activity in irradiated intestinal tissue, with E0703 showing greater effect. Group differences were evaluated using one‐way analysis of variance (ANOVA). ns, not significant; *p < 0.05; **p < 0.01; ***p < 0.001; and ****p < 0.0001.

To investigate whether E0703 alters the intestinal cellular microenvironment post irradiation and to elucidate the mechanism underlying E0703‐induced upregulation of intestinal Muc2, we used single‐cell RNA sequencing (scRNA‐seq) to profile the cellular landscape of the mouse small intestine under three conditions: control (CON), 5 days post irradiation (IR), and 5 days post irradiation with E0703 treatment (IR_Drug) (Figure 5A). In total, 18,951 cells were captured and classified into 12 cell types, including nine principal small intestinal populations (enterocyte, intestinal stem cell, transit amplifying [TA] cell, progenitor cell, goblet cell, enteroendocrine cell, Paneth cell, stromal cell, and endothelial cell) as well as three main immune cell types (B cell, T cell, and monocyte/macrophage) (Figure 5A,B). The intestinal microenvironment displayed marked differences between CON and IR groups. Post irradiation, the proportions of all three immune cell types were substantially decreased, whereas the proportions of enterocytes, stem cells, and goblet cells increased (Figure 5C). These findings are consistent with previous studies documenting the cellular dynamics of radiation‐induced intestinal injury^20^. In the IR_Drug group, both goblet and stem cell populations were further increased relative to the IR group (Figure 5D,E). Moreover, we observed significant upregulation of goblet cell marker genes, including *Muc2*, *Zg16*, and *Tff3,* and stem cell marker genes such as *Lgr5*, *Slc12a2*, and *Smoc2* in the IR_Drug group (Figures 5F and 6A). These results indicate that E0703 administration modulates the cellular composition of the irradiated small intestine and strongly influences the core biological functions of both stem cells and goblet cells.

Functional enrichment analysis of differentially expressed genes (DEGs) in stem cells across the three groups revealed distinct patterns. In the CON group, immune‐related pathways, such as T/B cell receptor signaling, were significantly enriched. In the IR group, protein synthesis and glutathione metabolism were prominent. In contrast, stem cells in the IR_Drug group showed notable activation of oxidative phosphorylation and ribosomal functions, as well as enrichment in the estrogen signaling pathway (Figures 6B and S3A). For goblet cells, immune‐related pathways, including antigen processing and presentation and the IL‐17 signaling pathway, were enriched in the CON group, whereas both irradiated states showed pronounced enrichment in ribosomal pathways (Figures 6C and S3B). Interestingly, the oxidative phosphorylation pathway was significantly activated in both stem cells and goblet cells in the IR_Drug group (Figure 6B,C). A suite of genes associated with oxidative phosphorylation, including *Atp5e, Cox8a, Cox6a1, Uqcr11, Uqcrq, Cox5b, Uqcr10, Cox7a2, Atp5j2, Ndufv3, Ndufa2, Ndufs6, Ndufa1, Ndufc1, and Atp5l*, was significantly upregulated in these cell types in the IR_Drug group (Figures 6D,E and S3C). In addition, E0703 improved mitochondrial function in the intestinal tissue of irradiated mice (Figure 6F–J), which is essential for oxidative phosphorylation. These results suggest that E0703 administration enhances intracellular energy metabolism in goblet and stem cells after irradiation. The ribosomal pathways in both cell types were also significantly activated, providing a functional basis for increased protein translation and assembly during tissue repair (Figure 6B,C). Following irradiation, cell–cell communication between immune cells and goblet/stem cells was markedly reduced compared to the CON group (Figure S4), indicating decreased immune‐related gene activity in these cell types. This reduction likely reflects radiation‐induced impairment of immune function. Collectively, these findings demonstrate that E0703 increases intestinal Muc2 expression in irradiated mice, an effect closely associated with enhanced oxidative phosphorylation activity in intestinal stem cells and goblet cells.

## DISCUSSION

The development of safe and reliable drugs for acute radiation protection, especially those effective at lethal doses with minimal side effects, remains a significant global challenge. Estrogen has received considerable attention as a potential mitigator of radiation‐induced damage. Beyond its role in reproductive health, estrogen can alleviate RI through its antioxidative properties, regulation of DNA damage, and modulation of inflammation^21^, ^22^. Estrogens act primarily via estrogen receptor 1 (Esr1) and Esr2, nuclear receptors that dimerize upon ligand binding to regulate gene expression by interacting with estrogen response elements^23^. E0703 preferentially activates Esr2 and, although less potent than estrogen itself, contributes to cell cycle regulation and DNA repair^15^, ^24^. Estrogenic therapies, including phytoestrogens, have been proposed for neuroprotection in non‐lethal radiation‐induced brain injury^16^. However, limited research has addressed estrogen's effect on radiation lethality and its possible radioprotective mechanisms involving gut microbiota modulation.

To simulate acute nuclear radiation exposure more accurately, we used a whole‐body irradiation model in mice, highlighting the critical effect of high‐dose radiation‐induced intestinal injury on survival. In this context, E0703 conferred marked protection against lethal 8.5 Gy irradiation, with a notable protective effect observed within 1 week after a single dose. Previous studies indicated that a single oral dose of E0703 results in detectable plasma concentrations for approximately 50 h in macaques^16^. Accordingly, we implemented a multiple dosing regimen, which further increased survival rates in mice following high‐dose irradiation.

This study focused on the importance of repairing intestinal injury after high‐dose acute whole‐body irradiation. The gut microbiota forms a stable symbiotic association with the host, critical for health maintenance. Disruption of this balance, particularly following radiation exposure, has been associated with the development of gastrointestinal diseases^25^. Radiation exposure can decrease beneficial bacteria and increase harmful bacteria, as indicated by reductions in *Lactobacillus* and *Bifidobacterium* species, with concurrent increases in *Escherichia coli* and *Staphylococcus* species, contributing to the onset and progression of radiation‐induced intestinal diseases (RIID)^26^, ^27^. Radiotherapy, although effective for tumor control, can damage healthy intestinal tissue, especially in the treatment of abdominal and pelvic malignancies, resulting in enteritis and significant shifts in the gut microbiome. These alterations are associated with increased morbidity, mortality, and decreased quality of life in both cancer patients and survivors^28^. Recent work has shown that fecal microbiota transplantation (FMT) can alleviate acute radiation syndrome, which is associated with increased levels of microbial‐derived indole‐3‐propionic acid in irradiated mice^29^.

Our findings revealed that E0703 treatment induced notable changes in the gut microbiota composition, particularly promoting a substantial increase in *AKK* abundance in irradiated mice. AKK is recognized as a probiotic that confers health benefits through the secretion of proteins, extracellular vesicles, and short‐chain fatty acids^30^, ^31^, ^32^, ^33^, ^34^. Specifically, AKK‐derived propionic acid acts on G‐protein‐coupled receptor 43 on intestinal epithelial cells, increasing histone acetylation and thereby enhancing the expression of tight junction proteins (Occludin, ZO‐1) and mucins. This strengthens the intestinal epithelial barrier and reduces radiation‐induced injury^35^. We demonstrated that AKK not only mitigates irradiation‐induced intestinal damage but also improves survival rates following irradiation. Although AKK administration maintained survival for only 1 week after irradiation, this may offer a valuable protective window for individuals acutely exposed to radiation, such as nuclear accident workers, where daily probiotic intake could support survival during critical periods. Notably, the combination of E0703 and AKK yielded the highest survival rates post irradiation, even with a modest 10% increase over either intervention alone, which could be crucial for increasing the likelihood of successful rescue in nuclear emergencies.

We further identified GlcN‐6‐P, produced by AKK, as a key metabolite supporting the repair of radiation‐induced intestinal damage. GlcN‐6‐P (C6H13NO8P) is synthesized from glucose and ATP by glucose kinase, serving as a precursor for amino sugar and polysaccharide synthesis and as a fundamental substrate for bacterial cell wall formation^36^. In cellular metabolism, GlcN‐6‐P can be converted into glucose and used for cellular energy. Radiation‐induced mitochondrial dysfunction disrupts glycolysis and other essential metabolic processes^37^. Direct supplementation with GlcN‐6‐P may restore energy metabolism in irradiated cells, thereby promoting recovery. Additionally, GlcN‐6‐P confers resistance to oxidative stress^36^, supporting cellular repair following RI. Other metabolites may also contribute to intestinal wall recovery and merit further investigation.

Few agents have been shown to induce AKK proliferation, with metformin, tryptophan derivatives, and betaine among those reported^38^, ^39^, ^40^. Although E0703 significantly increased AKK abundance *in vivo*, *in vitro* experiments did not demonstrate a direct proliferative effect, suggesting that E0703's influence on AKK is likely mediated through the intestinal microenvironment. We observed that E0703 promoted recovery of intestinal barrier stem cells and goblet cells post irradiation. Goblet cell‐derived mucin is an essential nutrient for AKK^41^. Muc2, the principal component of intestinal mucus, plays roles in signal transduction, cell adhesion, growth, and immune regulation, and forms the structural barrier that separates the microbiota from intestinal epithelial cells^42^, ^43^. By interacting with dendritic cells, Muc2 modulates immune responses, limits antigen immunogenicity, and protects epithelial cells from invasion, supporting intestinal homeostasis and immune tolerance^44^. Given the pivotal role of Muc2 in protecting the intestinal barrier and promoting AKK proliferation, we have focused on elucidating the mechanisms by which E0703 enhances Muc2 expression and secretion, thereby increasing AKK abundance.

Interestingly, our single‐cell transcriptome analysis revealed significant activation of the oxidative phosphorylation pathway in both intestinal stem cells and goblet cells. Oxidative phosphorylation is a fundamental biochemical process that takes place in the inner mitochondrial membrane of eukaryotic cells or in the cytoplasm of prokaryotes. It couples the energy released from the oxidation of substrates to the synthesis of ATP from ADP and inorganic phosphate via the respiratory chain^45^. Muc2 is synthesized by cytosolic ribosomes in goblet cells and undergoes a series of energy‐dependent modifications before being transported to the cell surface, released into the intestinal lumen, and forming a mucus gel together with water and other components^46^. This entire process requires substantial energy input from mitochondria, with oxidative phosphorylation being essential for ATP production. Notably, mitochondria are the only organelles in mammalian cells containing their own DNA, making them particularly susceptible to radiation‐induced damage, including double‐strand breaks, base mismatches, and fragment loss^47^. Unlike nuclear DNA, mitochondrial DNA lacks histone protection, resulting in greater vulnerability to damage and positioning mitochondria as critical targets in radiation‐induced cellular injury outside the nucleus^48^. Radiation disrupts mitochondrial function by impairing the aerobic respiratory chain, inducing oxidative stress, and activating the mitochondrial apoptotic pathway, ultimately leading to cell death. Meanwhile, radiation can damage genes encoding proteins of the aerobic respiratory chain, impairing ATP generation and jeopardizing cell survival^49^. Under normal conditions, electron leakage from the respiratory chain during aerobic respiration leads to the generation of reactive superoxide anions, the precursors of all reactive oxygen species (ROS). Upon radiation exposure, mitochondria become the primary site for excessive ROS production, which perpetuates oxidative stress and mitochondrial dysfunction. Therefore, mitochondrial oxidative stress and DNA damage are key aspects of radiation research^37^. E0703 effectively activates the oxidative phosphorylation pathway in intestinal stem cells and goblet cells, thereby providing critical energy required for intestinal barrier repair. The preservation of mitochondrial function by E0703 underlies the differentiation of stem cells into goblet cells and supports the production and secretion of mucin. In summary, E0703 promotes a beneficial feedback loop between intestinal epithelial cells and AKK, enhancing intestinal repair and improving survival rates following irradiation.

This study has certain limitations. First, although E0703 is an estrogen derivative, we did not include an estradiol intervention group as a control, as previous studies established E0703 as a more effective and less toxic radiation protectant compared with estrogen. Currently, Amifostine remains the only FDA‐approved drug for radiation protection, and it was included as the positive control in our experiments. Second, our research focused on the comprehensive effects of E0703 on radiation‐induced changes in intestinal cells and gut microbiota, particularly energy metabolism, but we did not explore potential novel targets beyond Esr2. This will be a subject of future research. Third, immune cells in the intestinal barrier play a crucial role in maintaining intestinal homeostasis; however, we did not conduct a detailed analysis of immune cell subpopulations following radiation exposure. Lastly, ethical constraints precluded the evaluation of E0703's radioprotective effect in a human cohort, but our study offers important references for future research and drug development in radiation protection.

In conclusion, lethal irradiation causes severe intestinal injury and disrupts the gut microbiota. Early intervention to repair intestinal damage and restore microbial balance can significantly enhance survival. E0703 is an effective oral radioprotective agent that improves mitochondrial function in intestinal stem and goblet cells, promotes mucin secretion, and supports intestinal repair. Additionally, E0703 increases AKK abundance post irradiation; AKK utilizes mucin and produces GlcN‐6‐P, which further aids in intestinal cell recovery. Collectively, E0703 directly benefits goblet cells and AKK, fostering a positive interaction between host intestinal cells and probiotics, and significantly increases survival rates in mice exposed to acute lethal irradiation.

## MATERIALS AND METHODS

### Mice and irradiation

Seven‐week‐old male C57BL/6J mice (approximately 21 g) were obtained from Beijing Vital River Laboratory Animal Technology Co., Ltd. Upon arrival, mice were acclimated for 1 week in a Specific Pathogen‐Free (SPF) facility at the Beijing Institute of Radiation Medicine (BIRM). Animals were maintained under controlled environmental conditions (22 ± 2°C, 40%–70% humidity, 12‐h light/dark cycle) with *ad libitum* access to standard chow and water.

To assess the radioprotective effect of E0703, mice were randomly assigned to four groups. The control group (CON, *n* = 10) consisted of healthy mice without irradiation or medication. The IR (*n* = 10) received a single whole‐body γ‐irradiation using a ^60^Co source at a source‐to‐skin distance of 250 cm, a dose rate of 70 R/min, and a total absorbed dose of 8.5 Gy. The IRA group (*n* = 10) was pretreated with Amifostine (150 mg/kg; MedChemExpress, HY‐B0639) via an intraperitoneal injection 0.5 h before irradiation. The IRB group (*n* = 10) was pretreated with E0703 (5 mg/kg, purity 99.96%; BIRM), administered orally in sterile corn oil once, 24 h before irradiation. All experiments were repeated in three independent replicates. Irradiated mice were monitored for 1 week, with daily recording of body weight and survival. On Day 5 after irradiation, feces were collected for gut microbiota analysis, and small intestine samples were collected for histopathology. To assess the effect of E0703 on gut microbiota, additional mice received a single oral gavage of E0703 (5 mg/kg), and feces were collected before administration and on Day 1, 5, and 10 after treatment. Fecal GlcN‐6‐P was measured using an enzyme‐linked immunosorbent assay (ELISA) Kit (Enzyme‐linked Biotechnology, MM‐92820001) according to the manufacturer's protocol.

### Hematoxylin–eosin (HE) staining and immunofluorescence staining

A 3 cm segment of the proximal small intestine was excised and fixed in 4% paraformaldehyde for 72 h. Fixed tissues were embedded, sectioned at 5 μm thickness, and stained with HE. For immunofluorescence, sections were deparaffinized, rehydrated, and subjected to antigen retrieval using EDTA buffer (pH 8.0). After blocking, sections were incubated overnight with primary antibodies: anti‐Muc2 (GB11344; Guge), anti‐Ki67 (GB121141; Guge), and anti‐Lyz (MA5‐32154; Thermo Fisher Scientific). After washing, sections were incubated with Cy3‐conjugated secondary antibodies (Goat Anti‐Rabbit Cy3, B100802; Goat Anti‐Mouse Cy3, B100801; Baiqiandu) for 1 h. TUNEL staining was performed with a TUNEL Kit (11684817910, Roche), using 50 μl of reaction solution on dewaxed sections for 2 h in the dark. Sections were counterstained with DAPI and analyzed using ImageJ software.

### 16S rDNA gene sequencing

Fecal DNA extraction, PCR amplification, Illumina sequencing, and sequence data processing followed protocols previously described^50^, ^51^, ^52^. The Silva database (threshold 70%) was used to determine taxonomic composition at different levels. Operational taxonomic units (OTUs) were analyzed to assess microbiota diversity and abundance in all groups. The sobs index, calculated using mothur software (v1.30.2), was used to reflect observed species richness. PCA was conducted with R (v3.3.1) to evaluate community composition and sample differences. Community bar charts at the phylum level and heatmaps at phylum and species levels were constructed using R (vegan package). Species differences among groups were analyzed using the Kruskal–Wallis H test and multiple test correction. LEfSe (http://huttenhower.sph.harvard.edu/galaxy/root?tool_id=lefse_upload) identified species with differential abundance, with LDA used to estimate species contributions to group differences.

### Detection of *A. muciniphila* in mice

For bacterial DNA extraction and quantitative PCR (qPCR), fecal DNA was extracted using the Stool Kit (Servicebio, G3638), following the manufacturer's protocol. DNA concentration was determined using a quantification assay. Specific primers for *A. muciniphila* (sense: CCCTGTCATGTGGGAGCAAAT; antisense: AACAGCCTACGCACGCTTTAC) were used. qPCR was performed using a SYBR qPCR Master Mix kit with an initial denaturation at 95°C for 3 min, followed by 40 cycles of 95°C for 15 s, 60°C for 30 s, and 72°C for 30 s. Relative gene expression was determined using the $2^{-∆∆C_{t}}$ method.

### *Akkemansia muciniphila* culture and drug intervention

*A. muciniphila* (BNCC341917, BeNa Culture Collection) was cultured in fluid thioglycollate medium at 37°C under anaerobic conditions. E0703, dissolved in dimethyl sulfoxide (DMSO), was added at concentrations of 0, 15, 30, 60, and 120 ng/ml for 48 h, and bacterial growth was evaluated by measuring optical density (OD) at 600 nm. Recombinant human mucin 2 (KL‐R20237Hu; Kang Lang Biological) was added to the medium at 20, 10, and 5 μg/ml. The relative abundance of AKK was determined by measuring OD at 600 nm after 24, 48, and 72 h.

### *A. muciniphila* intervention

Mice were fasted for 8 h prior to irradiation. Beginning 10 days before irradiation, the IRAK and IRBAK groups received daily AKK at 5 × 10^7^ colony‐forming units (CFU)/kg by oral gavage. The IRB and IRBAK groups were administered E0703 at 5 mg/kg in corn oil daily by oral gavage, whereas the IRA group received Amifostine intraperitoneally at 150 mg/kg. The CON and IR groups received 10 ml/kg corn oil as vehicle controls. Each group comprised 10 mice. All treatments were administered daily for the duration of the intervention period. γ‐Radiation was delivered using a ^60^Co source (BIRM, Beijing Institute of Radiation Medicine). Except for the CON group, all mice were placed in a radiation box and subjected to a single whole‐body irradiation at a source‐to‐skin distance of 250 cm, a dose rate of 70 R/min, and a total dose of 8.5 Gy to induce radiation‐related intestinal injury. Mice that showed severe clinical signs or abdominal aortic aneurysm were humanely euthanized by an intraperitoneal injection of pentobarbital sodium (100 mg/kg).

### Immunohistochemistry and PAS staining

Primary antibodies against Lgr5 (AF0165; Beyotime Biotechnology) and Olfm4 (DF13440; Affinity Biosciences) were used for immunohistochemical staining. After incubation with the secondary antibody at 37°C for 30 min, samples were rinsed three times with PBS and counterstained with hematoxylin. Sections were then dehydrated and mounted for microscopic analysis. Lgr5‐positive areas were quantified using ImageJ software by analyzing five random fields per section. Mean values were calculated for statistical analysis. For PAS staining, deparaffinized sections were incubated in periodic acid solution for 15 min, rinsed, and then immersed in Schiff's reagent for 30 min in the dark. Following additional rinsing, sections were stained with hematoxylin, dehydrated, and sealed for examination.

### Intestine organoid culture and fecal supernatant intervention

Lgr5‐EGFP‐IRES‐creERT2 (Lgr5‐GFP) mice (Cyagen Biosciences) were used for crypt isolation and organoid culture. Small intestines were harvested, washed with PBS, and segmented. Crypts were isolated by incubating the segments in 2.5 mM EDTA at 4°C for 40 min. The supernatant was discarded, and the tissue was resuspended in PBS containing 0.1% BSA at 4°C, filtered through a 70 μm cell strainer, and centrifuged at 290 g for 5 min. After a wash with PBS/BSA, the pellet was centrifuged at 200*g* for 5 min to obtain a single‐cell suspension. For culture, 500 crypts were suspended in a 1:1 mixture of IntestiCult Organoid Growth Medium (06005; Stemcell) and Matrigel (356237; Corning), and 50 μl of this mixture was added to the center of each well in a 24‐well plate. After Matrigel solidification, 500 μl of growth medium supplemented with the respective group's fecal supernatant was added.

To prepare the fecal supernatant, 100 mg of mouse cecal contents were diluted in 1 ml of PBS, shaken on ice for 1 h, and gently swirled. Samples were centrifuged at 4000 rpm for 10 min, and the supernatant was filtered through a 0.22 μm filter. For organoid culture, the medium contained 0.01% (v/v) fecal supernatant. Medium was refreshed every 72 h. Organoid surface areas were measured on Day 1, 3, and 5 using an inverted microscope (Cytation5; BioTek) and quantified using ImageJ software.

### Irradiation of intestinal organoids and drug intervention

Intestinal organoids were irradiated with 8.5 Gy at 24 h after seeding, at a dose rate of 0.69 R/min. One group received E0703 (60 ng/ml) prior to irradiation, whereas the control group received an equivalent concentration of DMSO. An additional group was treated with Esr2 antagonist 4‐[2‐Phenyl‐5,7‐bis(trifluoromethyl)pyrazolo[1,5‐a]pyrimidin‐3‐yl]phenol (PHTPP) (1 μM, MCE, HY‐103456) in combination with E0703 (60 ng/ml). A fourth group was treated with GlcN‐6‐P (10 μM, G5509; Merck) before irradiation. Organoid surface areas were analyzed on Day 1, 3, and 5 using an inverted microscope (Cytation5; BioTek) and quantified with ImageJ.

### Sample preparation for metabolome and transcriptome sequencing

AKK was cultured in fluid thioglycollate medium at 37°C under anaerobic conditions. For the intervention, E0703 was dissolved in DMSO and added to the medium at 60 ng/ml (Drug group). The blank group contained only culture medium; the AKK group contained only bacteria; and the DMSO group included AKK with DMSO equivalent to the drug group. After 48 h of culture, the medium was centrifuged at 7000*g* for 15 min to collect both the supernatant and the AKK pellet for subsequent metabolomic and transcriptomic analyses.

### Prokaryotic transcriptome analysis

Transcriptome sequencing was performed using sediment from the AKK, DMSO, and drug groups. For library construction, the TruSeq™ Stranded Total RNA Library Prep Kit was used, in which dUTP replaced dTTP during second‐strand cDNA synthesis to facilitate strand specificity by incorporating A/U/C/G bases. The second cDNA strand was subsequently digested with uracil‐N‐glycosylase (UNG), ensuring that the library retained only the first strand. Total RNA was extracted from tissue samples, and its concentration and purity were assessed using a NanoDrop 2000. RNA integrity was verified by agarose gel electrophoresis and quantified using the Agilent 2100 system to determine RNA integrity number (RIN) values. Each library required a minimum of 2 μg of total RNA with a concentration ≥100 ng/μl and an OD_260/280_ ratio between 1.8 and 2.2. As prokaryotic mRNA lacks the 3′ polyA tail present in eukaryotic transcripts, rRNA depletion was conducted to enrich mRNA. Because the Illumina platform sequences short fragments, the enriched mRNA, typically several kilobases in length, was randomly sheared into ~200 bp fragments. Single‐stranded cDNA was synthesized using random primers and reverse transcriptase. During second‐strand synthesis, dUTP replaced dTTP to mark the strand for degradation. The resulting double‐stranded cDNA, initially with sticky ends, was blunt‐ended using End Repair Mix, followed by the addition of an A base to the 3’ end for ligation of Y‐shaped adaptors. UNG digestion was again used to remove the second strand prior to amplification. Library enrichment was carried out by 15 cycles of PCR, followed by quantification using the TBS380 fluorometer (PicoGreen assay). Libraries were pooled based on concentration and sequenced using the Illumina HiSeq platform with 2 × 150 bp or 2 × 300 bp paired‐end reads, after cluster generation by bridge PCR on the cBot. Downstream data analysis was performed in R. Differential gene expression heatmaps were generated using the ClusterGVis package. For functional enrichment analysis, gene IDs were converted into KEGG Orthology terms using the clusterProfiler package, and KEGG pathway enrichment was then conducted.

### Untargeted metabolomics of bacterial supernatant

Supernatant samples from the four groups were processed as follows: 50 μl of supernatant was transferred to a 1.5 ml EP tube at 4°C, 200 μl of cold methanol (containing internal standards; see Table S1) was added, vortexed for 2 min, and left at low temperature for 10 min. After centrifugation at 14,000*g* for 15 min at 4°C, 200 μl of supernatant was transferred to a new tube, concentrated by low‐temperature centrifugation, and stored at –20°C. Before analysis, extracts were reconstituted in 100 μl of 20% methanol/water, vortexed, centrifuged, and transferred to vials for positive and negative ion analyses. For chromatographic separation, a Beh C8 column (1.7 μm, 2.1 × 100 mm) was used for positive mode and an HSS T3 column (1.8 μm, 2.1 × 100 mm) was used for negative mode; column temperature 50°C, injection volume 5 μl, flow rate 0.35 ml/min, and mobile phases of 0.1% formic acid/water (A) and 0.1% formic acid/acetonitrile (B). The gradient was 0–1 min, 5% B; 1.1–11 min, 5–100% B; 11.1–13 min, 100% B; 13.1–15 min, 5% B. Mass spectrometry was performed using heated electrospray ionization (HESI)‐positive and ‐negative modes with full scan and data‐dependent acquisition (DDA); settings included Spray Voltage +3.8 kV, capillary temperature 320°C, aux gas heater 350°C, sheath gas 35 Arb, aux gas 8 Arb, S‐lens RF 50, *m*/*z* 70–1050, full MS resolution 70,000, MS/MS resolution 17,500, TopN 5, and stepped NCE 20/40. Data were visualized in R (v4.3.1).

### Single‐cell transcriptome library construction and the sequencing process

Single‐cell suspensions were assessed for viability (>85%) and concentration (700–1200 cells/μl). Qualified samples were loaded into Chromium Chip G (10× Genomics), where cells, gel beads, and oil were partitioned into gel beads in emulsion (GEMs) via microfluidics. Gel beads released barcoded primers, and cell lysis released mRNA, which was reverse‐transcribed to first‐strand cDNA containing 10× Barcodes and unique molecular identifiers (UMIs). After GEM breakage, cDNA was purified and amplified by PCR. Amplified cDNA was fragmented, and 200–300 bp fragments were selected. Libraries were constructed using end repair, A‐tailing, and adaptor ligation for sequencing on the Illumina platform (Annoroad Gene Technology).

### Single‐cell RNA‐seq data analysis

Reads were mapped to the mouse reference genome (mm10) using Cellranger (v7.0.0) to generate a UMI‐based gene expression matrix. Data were filtered, normalized, integrated, and clustered in Seurat (v4.3.0.1)^53^ as previously described^54^, ^55^, ^56^. Initial quality control excluded genes detected in fewer than three cells, cells expressing fewer than 200 genes, and cells with >30% mitochondrial reads; doublets were removed using DoubletFinder (v2.0.4)^57^. The final dataset comprised 19,614 genes from 18,951 cells. Data were subjected to log normalization and scaling (*NormalizeData*, *ScaleData* functions), and canonical correlation analysis (CCA) was used to integrate datasets. Clustering (*FindClusters*) and uniform manifold approximation and projection (UMAP) were used for dimensionality reduction. Signature genes for each cell type were identified using *FindAllMarkers*. DEGs (adjusted *p* < 0.05, log_2_(fold change) > 0.25) were subjected to gene enrichment analysis with clusterProfiler (v4.8.3)^58^. Cell–cell communication was analyzed using CellChat (v2.1.2)^59^, and CCCs with fewer than 10 cells per cell type were excluded.

### Detection of the mitochondrial respiratory chain

Fresh small intestine tissues were collected from mice. For each sample, 1 g of tissue was homogenized in 9 ml of PBS (pH 7.2–7.4) and centrifuged at 3000 rpm for 20 min. The supernatant was collected and processed according to the kit instructions (Jiangsu Meimian Industrial Co., Ltd.). Activities of mitochondrial respiratory chain complexes I (MM‐44745M1), II (MM‐44757M1), III (MM‐44948M1), IV (MM‐47091M1), and V (MM‐45014M1) were assayed.

### Statistical analysis

Statistical analysis was performed using SPSS v19.0 (IBM). Data are presented as mean ± standard deviation (SD). Statistical significance was set at *p* < 0.05. Group differences were evaluated using one‐way analysis of variance (ANOVA), with post hoc Tukey tests for homogeneous variances and Games–Howell tests for heterogeneous variances. Survival analysis was performed using Kaplan–Meier curves, with log‐rank (Mantel–Cox) and Gehan–Breslow–Wilcoxon tests for pairwise comparisons. GraphPad Prism v9 (GraphPad Software) was utilized for graphical visualization.

## AUTHOR CONTRIBUTIONS

**Zhexin Ni**: Investigation; software; validation; visualization; writing—original draft; writing—review and editing. **Ziqiao Yan**: Investigation; software; validation; visualization; writing—original draft. **Mingyang Chang**: Data curation; investigation; methodology; software; visualization; writing—original draft. **Yangshuo Li**: Data curation; investigation; software; visualization; writing—original draft. **Tiantian Xia:** Validation, Investigation. **Zebin Liao:** Validation, Investigation. **Zhijie Bai:** Validation, Investigation. **Ningning Wang:** Validation, Investigation. **Chaoji Huangfu:** Investigation, Methodology. **Dezhi Sun:** Investigation, Methodology. **Yangyi Hu:** Visualization, Methodology. **Liangliang Zhang:** Validation, Investigation. **Feiran Hao:** Investigation, Methodology. **Yongqi Dou:** Investigation, Methodology. **Pan Shen**: Data curation; methodology; software; supervision; writing—original draft. **Wei Zhou**: Conceptualization; project administration; supervision; writing—review and editing. **Yue Gao**: Conceptualization; funding acquisition; project administration; supervision; writing—review and editing.

## ETHICS STATEMENT

All experimental procedures involving animals were conducted in accordance with the ethical standards and guidelines approved by the Animal Care and Ethics Committee of BIRM (approval number: IACUC‐DWZX‐2020‐783), following the Guide for the Care and Use of Laboratory Animals and the National Institutes of Health guidelines.

## CONFLICT OF INTERESTS

The authors declare no conflict of interests.

## Supporting information

Supporting information.

Supporting information.

Supporting information.

Supporting information.

Supporting information.

## Data Availability

All data are available in the main text or the supplementary materials. The datasets generated during and/or analyzed during the current study are available in the China National GeneBank DataBase repository (https://db.cngb.org), including single‐cell transcriptome (CNP0006034) and gut flora sequencing (CNP0006026). Metabolome (PRJCA028681) are available in the China National Center for Bioinformation repository (https://www.cncb.ac.cn).
