# Supplementary figures and images for: The synthetic estradiol analog E0703 enhances Akkermansia muciniphila growth for radiation‐induced intestinal damage repair
Source: mLife. 2026 Apr 30;5(2):199–216. doi: 10.1002/mlf2.70071 (PMC13131332; doi:10.1002/mlf2.70071)

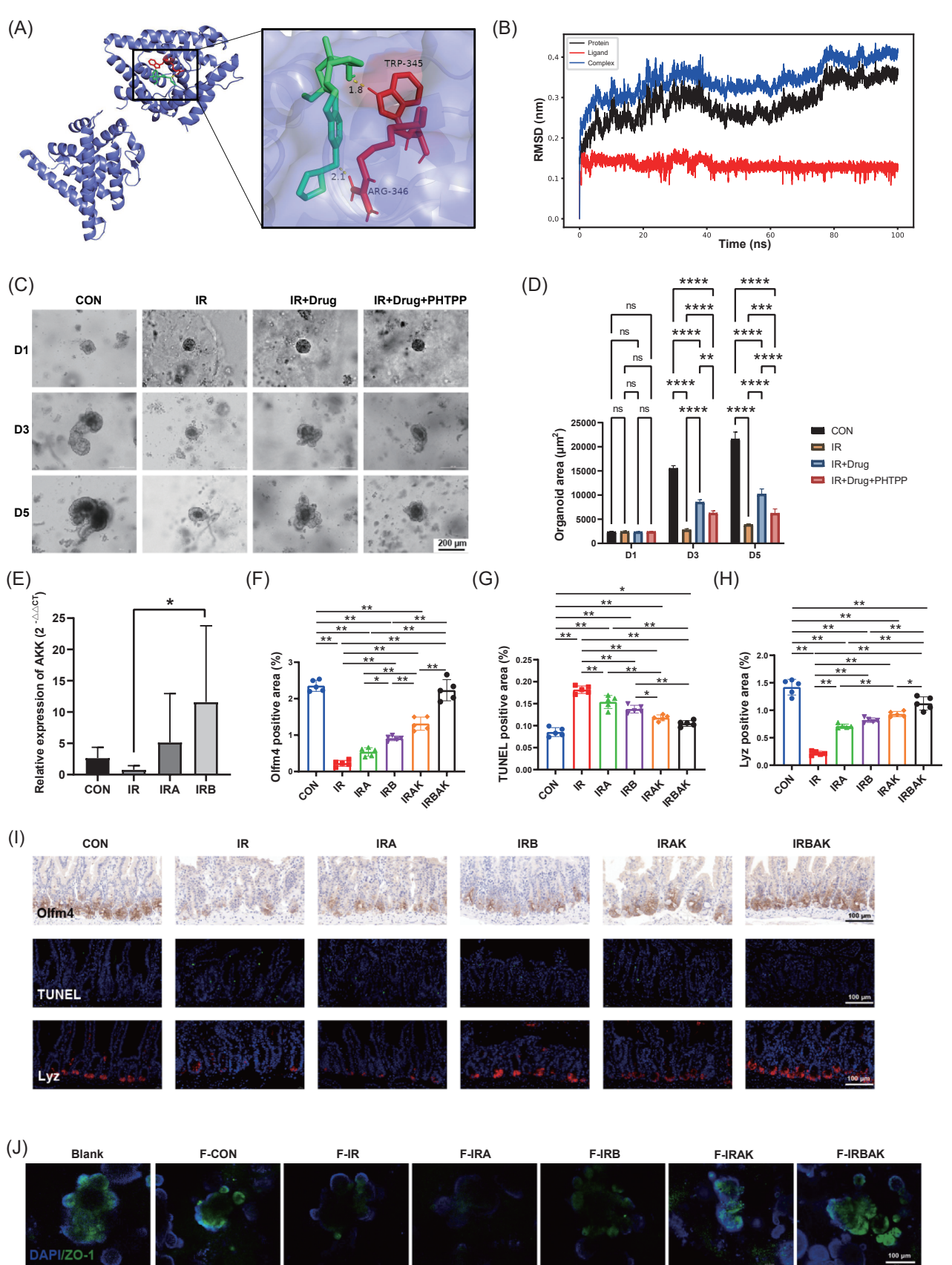

Supplement: Supplementary file 1 — Supporting information. [file MLF2-5-199-s005.pdf]

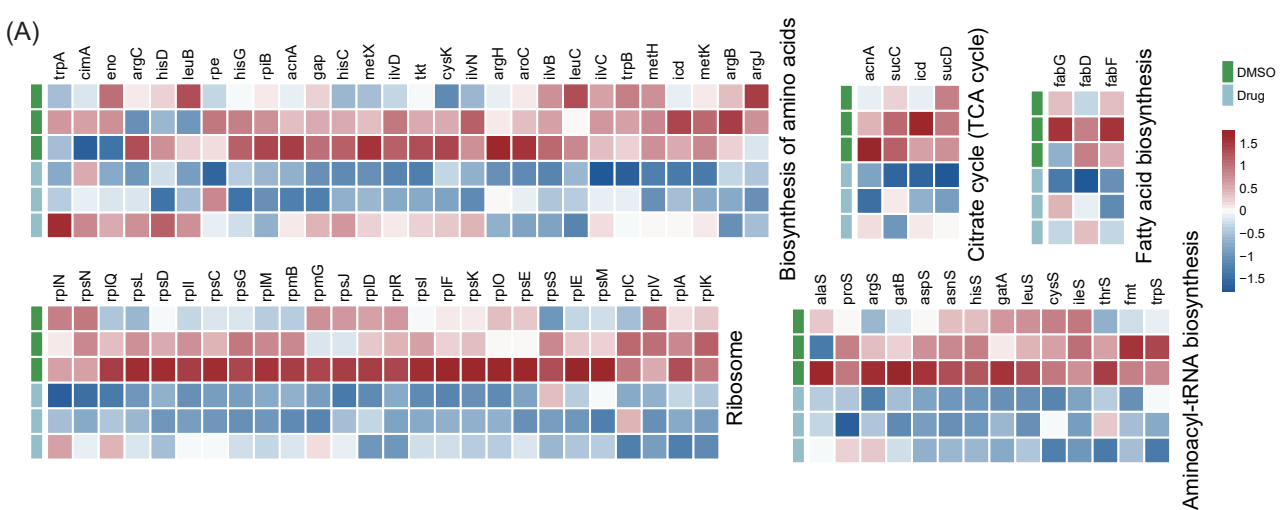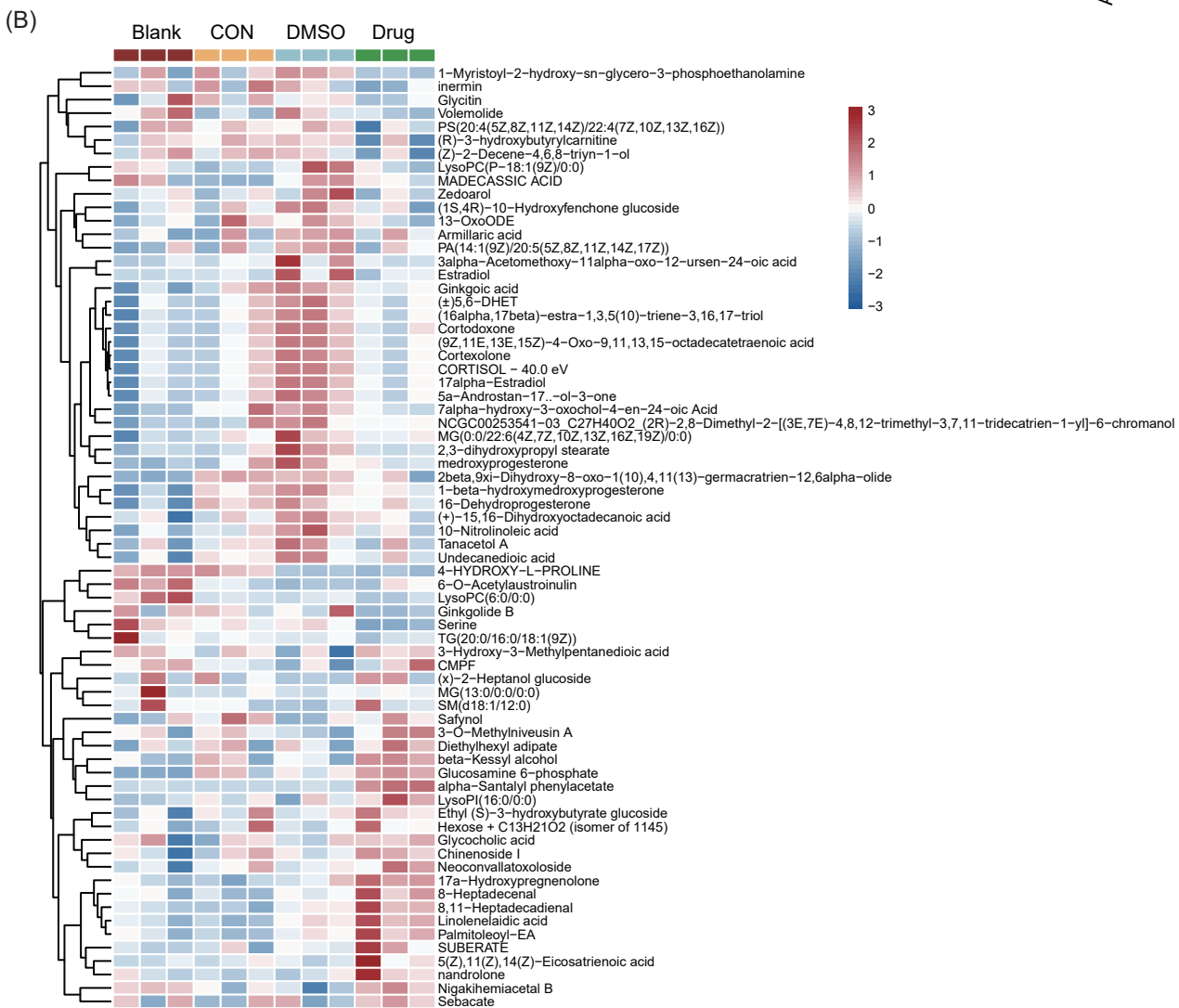

Supplement: Supplementary file 2 — Supporting information. [file MLF2-5-199-s004.pdf]

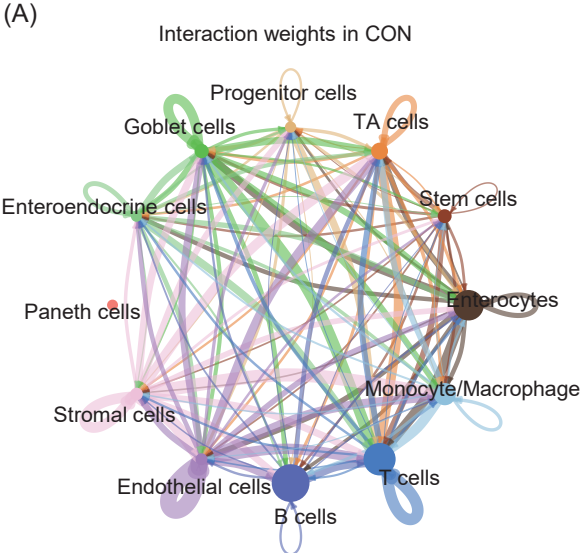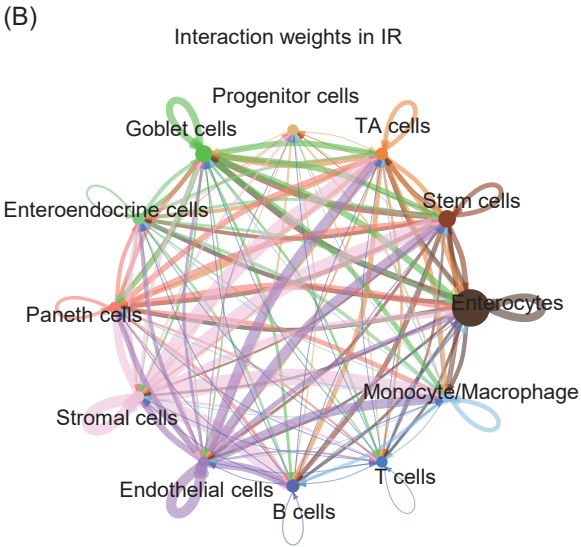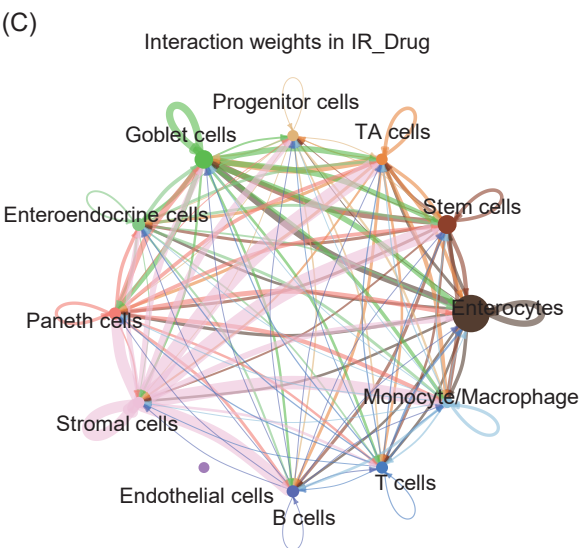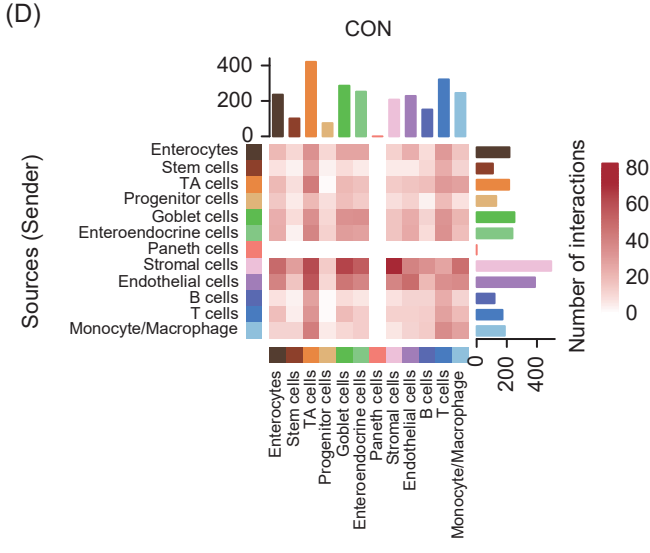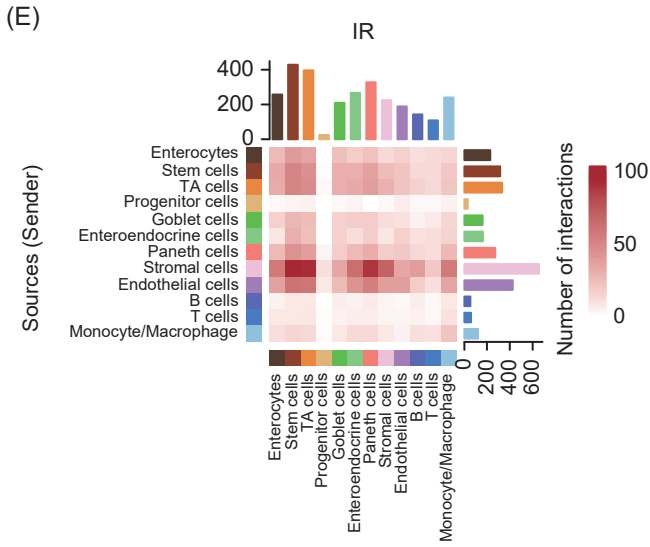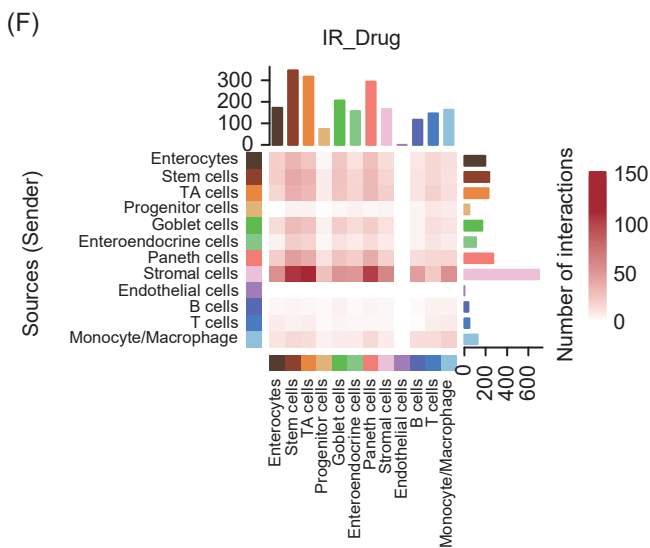

Supplement: Supplementary file 4 — Supporting information. [file MLF2-5-199-s002.pdf]
