# Supplementary material for: The synthetic estradiol analog E0703 enhances Akkermansia muciniphila growth for radiation‐induced intestinal damage repair
Source: mLife. 2026 Apr 30;5(2):199–216. doi: 10.1002/mlf2.70071 (PMC13131332; doi:10.1002/mlf2.70071)
Supplement: Supplementary file 3 — Supporting information. [file MLF2-5-199-s001.pdf]

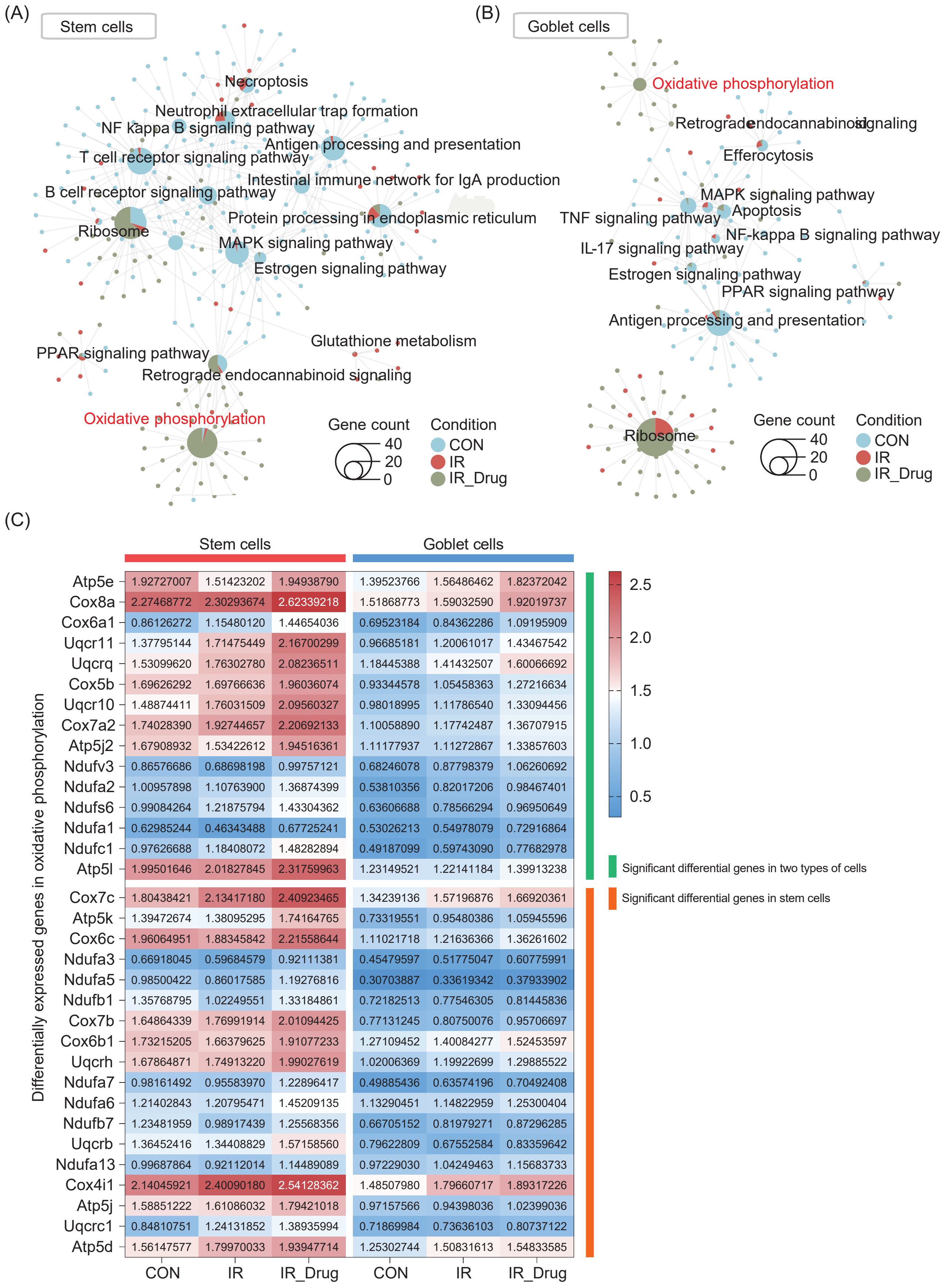
(A)
Stem cells
Necroptosis
Neutrophil extracellular trap formation
NF kappa B signaling pathway
Antigen processing and presentation
T cell receptor signaling pathway
Intestinal immune network for IgA production
B cell receptor signaling pathway
Protein processing in endoplasmic reticulum
Ribosome
MAPK signaling pathway
Estrogen signaling pathway
Glutathione metabolism
PPAR signaling pathway
Retrograde endocannabinoid signaling
Oxidative phosphorylation
Gene count
40
20
0
Condition
CON
IR
IR_Drug
(B)
Goblet cells
Oxidative phosphorylation
Retrograde endocannabinoid signaling
Efferocytosis
MAPK signaling pathway
Apoptosis
TNF signaling pathway
NF-kappa B signaling pathway
IL-17 signaling pathway
Estrogen signaling pathway
PPAR signaling pathway
Antigen processing and presentation
Ribosome
Gene count
40
20
0
Condition
CON
IR
IR_Drug
(C)
Stem cells
Goblet cells
Differentially expressed genes in oxidative phosphorylation
Atp5e 1.92727007 1.51423202 1.94938790 1.39523766 1.56486462 1.82372042
Cox8a 2.27468772 2.30293674 2.62339218 1.51868773 1.59032590 1.92019737
Cox6a1 0.86126272 1.15480120 1.44654036 0.69523184 0.84362286 1.09195909
Uqcr11 1.37795144 1.71475449 2.16700299 0.96685181 1.20061017 1.43467542
Uqcrq 1.53099620 1.76302780 2.08236511 1.18445388 1.41432507 1.60066692
Cox5b 1.69626292 1.69766636 1.96036074 0.93344578 1.05458363 1.27216634
Uqcr10 1.48874411 1.76031509 2.09560327 0.98018995 1.11786540 1.33094456
Cox7a2 1.74028390 1.92744657 2.20692133 1.10058890 1.17742487 1.36707915
Atp5j2 1.67908932 1.53422612 1.94516361 1.11177937 1.11272867 1.33857603
Ndufv3 0.86576686 0.68698198 0.99757121 0.68246078 0.87798379 1.06260692
Ndufa2 1.00957898 1.10763900 1.36874399 0.53810356 0.82017206 0.98467401
Ndufs6 0.99084264 1.21875794 1.43304362 0.63606688 0.78566294 0.96950649
Ndufa1 0.62985244 0.46343488 0.67725241 0.53026213 0.54978079 0.72916864
Ndufc1 0.97626688 1.18408072 1.48282894 0.49187099 0.59743090 0.77682978
Atp5l 1.99501646 2.01827845 2.31759963 1.23149521 1.22141184 1.39913238
Cox7c 1.80438421 2.13417180 2.40923465 1.34239136 1.57196876 1.66920361
Atp5k 1.39472674 1.38095295 1.74164765 0.73319551 0.95480386 1.05945596
Cox6c 1.96064951 1.88345842 2.21558644 1.11021718 1.21636366 1.36261602
Ndufa3 0.66918045 0.59684579 0.92111381 0.45479597 0.51775047 0.60775991
Ndufa5 0.98500422 0.86017585 1.19276816 0.30703887 0.33619342 0.37933902
Ndufb1 1.35768795 1.02249551 1.33184861 0.72182513 0.77546305 0.81445836
Cox7b 1.64864339 1.76991914 2.01094425 0.77131245 0.80750076 0.95706697
Cox6b1 1.73215205 1.66379625 1.91077233 1.27109452 1.40084277 1.52453597
Uqcrh 1.67864871 1.74913220 1.99027619 1.02006369 1.19922699 1.29885522
Ndufa7 0.98161492 0.95583970 1.22896417 0.49885436 0.63574196 0.70492408
Ndufa6 1.21402843 1.20795471 1.45209135 1.13290451 1.14822959 1.25300404
Ndufb7 1.23481959 0.98917439 1.25568356 0.66705152 0.81979271 0.87296285
Uqcrb 1.36452416 1.34408829 1.57158560 0.79622809 0.67552584 0.83359642
Ndufa13 0.99687864 0.92112014 1.14489089 0.97229030 1.04249463 1.15683733
Cox4i1 2.14045921 2.40090180 2.54128362 1.48507980 1.79660717 1.89317226
Atp5j 1.58851222 1.61086032 1.79421018 0.97157566 0.94398036 1.02399036
Uqcrc1 0.84810751 1.24131852 1.38935994 0.71869984 0.73636103 0.80737122
Atp5d 1.56147577 1.79970033 1.93947714 1.25302744 1.50831613 1.54833585
CON
IR
IR_Drug
CON
IR
IR_Drug
2.5
2.0
1.5
1.0
0.5
Significant differential genes in two types of cells
Significant differential genes in stem cells
