# Supplementary material for: The synthetic estradiol analog E0703 enhances Akkermansia muciniphila growth for radiation‐induced intestinal damage repair
Source: mLife. 2026 Apr 30;5(2):199–216. doi: 10.1002/mlf2.70071 (PMC13131332; doi:10.1002/mlf2.70071)
Supplement: Supplementary file 5 — Supporting information. [file MLF2-5-199-s003.docx]

**Supplementary figures**

**Supplementary figure 1. *Akkermansia muciniphila* supplementation reduces intestinal injury caused by irradiation. (A)** Molecular docking between E0703 and estrogen receptor 2. (**B**) Root Mean Square Deviation (RMSD) in the molecular docking between E0703 and estrogen receptor 2. Lower RMSD values here (＜0.4 nm) indicate more similarity between structures. (**C**) Representative photos of intestinal organoids in four groups. PHTPP is the estrogen receptor 2 antagonists. (**D**) Quantification of intestinal organoids growth status in four groups. (**E**) Quantification of the relative expression levels of the target genes in *Akkermansia muciniphila* among four groups through the application of the 2^-△△CT^ method. (**F-H**) Quantification of pathological indicators of Olfm4, TUNEL and Lyz. (**I**) Immumohistochemical and immunofluorescence staining of mice intestine after irradiation. Bars represent 100 μm (40×). (**J**) Representative images of ZO-1 immunofluorescence staining of intestinal organoids following intervention with fecal supernatants from six groups. *, *P*<0.05; **, *P*<0.01; ***, *P*<0.001; ****, *P*<0.0001.

**Supplementary figure 2. Analysis of the transcriptome and metabolome profiling of *Akkermansia muciniphila*. (A)** Heatmap illustrating the clustering of differentially expressed genes enriched in KEGG pathways between DMSO and Drug groups. **(B)** Heatmap displaying the major differential metabolites among four groups. The right-side displays metabolites with relatively lower or higher abundance in the E0307 group. Blank group, the normal culture medium; CON group, AKK without any intervention; DMSO group, AKK with dimethyl sulfoxide (DMSO) intervention; Drug group, AKK with E0703 (dissolved in DMSO) intervention.

**Supplementary figure 3. The functional changes across there conditions in stem cells and goblet cells.** (**A and B**) The pathway-gene network showing the functional changes occurring in stem and goblet cells under varying conditions. The point sizes represented the number of differentially expressed genes (DEGs) across various KEGG pathways. The pie chart represented the distribution of DEGs originating from different conditions. (**C**) Heatmap of the differentially expressed genes levels in stem and goblet cells under varying conditions.

**Supplementary figure 4. The cell-cell communication in mouse small intestine under varying conditions.** (**A-C**) The circular plot illustrating the overall cell-cell communication in the small intestine of the CON, IR and IR_Drug group. Thick lines represent interaction weights, while the size of the dots represents the cell counts. (**D-F**) The heatmap displaying the quantity of cell-cell communication in the small intestine among the CON, IR, and IR_Drug group. Darker colors indicate higher quantities of communication.

**Supplementary Table 1. The internal standard compound in untargeted metabolomics.**

| **NO.** | **Internal standard compound** | **Concentration (μg/mL)** |
| --- | --- | --- |
| 1 | Carnitine C2:0-d3 | 0.08 |
| 2 | Carnitine C8:0-d3 | 0.05 |
| 3 | Carnitine C10:0-d3 | 0.05 |
| 4 | Carnitine C16:0-d3 | 0.075 |
| 5 | LPC 19:0 | 0.375 |
| 6 | FFA C16:0-d3 | 1.25 |
| 7 | FFA C18:0-d3 | 1.25 |
| 8 | CDCA-d4 | 0.75 |
| 9 | CA-d4 | 0.925 |
| 10 | Trp-d5 | 2.125 |
| 11 | Phe-d5 | 1.8 |
| 12 | SM 12:0 | 0.375 |
| 13 | Choline-d4 | 1 |
